# Talented but Not Chosen—A Cross-Sectional Study—Do Coaches Release Late-Maturing Under-14 Players When Making Decisions?

**DOI:** 10.3390/jfmk10020127

**Published:** 2025-04-10

**Authors:** Fabiana Parreira Bonito, Júlia Teles, Tiago Matos, Filipe Jesus, Anna Volossovitch, Carlos Barrigas, Maria Isabel Fragoso

**Affiliations:** 1 Sports and Health Department, Interdisciplinary Center of Human Performance (CIPER), Faculty of Human Kinetics, University of Lisbon, 1649-004 Lisboa, Portugal; fabianabonito@fmh.ulisboa.pt; 2 Mathematical Methods Unit, CIPER, Faculty of Human Kinetics, University of Lisbon, 1649-004 Lisboa, Portugal; jteles@fmh.ulisboa.pt; 3 Sports and Health Department, Faculty of Human Kinetics, University of Lisbon, 1649-004 Lisboa, Portugal; tmatos@fmh.ulisboa.pt (T.M.); anavol@fmh.ulisboa.pt (A.V.); 4 Portugal Football School, Federação Portuguesa de Futebol, 1495-433 Cruz Quebrada-Dafundo, Portugal; 5 Exercise and Health Laboratory, CIPER, Faculty of Human Kinetics, University of Lisbon, 1649-004 Lisboa, Portugal; fastj96@gmail.com; 6 Physical Education Department, Agrupamento de Escolas das Laranjeiras, 1600-136 Lisboa, Portugal; cbarrigas@fmh.ulisboa.pt

**Keywords:** youth football, maturation, relative age effect, training age, skeletal age

## Abstract

**Background**: This cross-sectional study aims to clarify the relationship between coaches’ decisions and players’ relative age, biological maturation, and experience when identifying Under-14 (U14) players for the District and National Teams. **Methods**: A total of 360 male soccer players participating in the U14 national inter-association tournament, Lopes da Silva, were assessed. Birth dates were recorded, and players were categorized by birth quarter. Anthropometric measurements, fitness level (handgrip strength), and success indicators (performance evaluated by the coach, minutes played, and selection for the National Team) were documented, Maturation status was determined using bone age. **Results**: The majority of the sample was born in the 1st and 2nd quartiles regardless of maturation status. Morphological and functional differences between players born in different quartiles were no longer significant after controlling the effect of bone age. Late-maturing athletes played significantly fewer minutes. Among the players selected for the National Team, 89.9% were early or on-time maturers, and 92.9% were born in the first or second quartile of the year. While previous studies have shown reduced variability between bone and decimal age in the third and fourth quartiles, our findings indicate greater variability across all birth quartiles. **Conclusions**: Maturation status and relative age effect are crucial factors influencing coaches’ selection decisions. These findings highlight the need for a more equitable approach to talent identification, which should include track maturation bias avoiding the pursuit of short-term success and promoting long-term development programs for young soccer players. Undoubtedly, maturation status and relative age effect (RAE) play a significant role in a coach’s decision-making process.

## 1. Introduction

At present, talent identification is a major concern for most soccer clubs. The talent identification process facilitates better resource management (financial, equipment, human resources) and yields a greater return on investment [1]. Numerous scientific publications have suggested that talent identification should consider maturation, the relative age effect (RAE), and the training age [2,3]. Nevertheless, a winner-oriented environment has influenced talent identification and development programs to prioritize an athlete’s survival characteristics [4,5,6], particularly in professional clubs.

Maturity is defined as a state throughout a long maturation process which ends when adulthood, or any other final state considering all the tissues, organs, and systems, or the limit of their functional capabilities, has been reached at a certain age, or whenever documented standard references allow us to make comparisons [7]. The maturation time presents great individual variability, particularly during adolescence. In men’s soccer, early-maturing athletes are generally at a physical and functional advantage compared to late-maturing athletes of the same age, performing better in tasks related to strength, power, speed, agility, or endurance [2,8].

The RAE is the difference observed between athletes in the same age group category, where athletes born in January can be 12 months older than athletes born in December [9,10]. Chronologically older athletes will certainly be at an advantage, because they will be more experienced and, in most cases, will have larger body dimensions [7]. Although RAE is well-documented, it continues to impact most youth teams, with an overrepresentation of athletes born at the beginning of the selection year [8,11].

Training age refers to an athlete’s history of training, encompassing their experience in their current sport as well as any prior training activities [3]. It is a crucial factor to consider when prescribing training loads and developing both declarative and procedural knowledge [12].

Although they are two different topics, relative age effect (RAE) and maturation status (MS), interrelate with each other [13,14]. An athlete born at the end of the year may accidentally have advanced maturation, thereby managing to improve or even resolve the disadvantages that his relative age could cause. The opposite can also occur; athletes can be younger and additionally present late maturity, increasing their functional disadvantage in relation to their peers. On the other hand, the older athletes will have more experience than their peers, despite their maturation status [15]. Fragoso, et al. [16] observed that elite soccer athletes born late in the year have a greater bone age variability than their older peers, showing that athletes born early are more often on-time or maturing earlier than the younger ones.

Despite the complexities of the selection process and its crucial role in success, the responsibility for identifying talent in the early stages of development largely depends on coaches’ subjective opinions, shaped by their experiences, personal interpretations of elite soccer, and the training culture in which they operate [17,18]. Although the scientific community has long advocated for multidimensional models, coach evaluations and motor performance tests remain prevalent. This favors the selection and development of early-maturing players [2,4,8]. Conversely, physically disadvantaged players who manage to progress in elite academies often exhibit significantly higher technical and skill levels than their peers. They are also much more likely to secure professional contracts compared to their older teammates and have a greater propensity to transition from youth soccer to senior professional soccer. These findings reinforce the “underdog hypothesis” [19], highlighting that a viable pathway to selection remains open for late-maturing players [20].

Although this topic is not new, it is specific to each sporting level and the training culture in which scouting departments operate. Therefore, it seems essential to clarify the actual selection opportunities offered in elite youth soccer in Portugal. Consequently, the aims of this study were: (1) to confirm whether the interrelation between the RAE and MS, in elite teams, remains the same as that observed 10 years ago [16]; (2) to understand if the coaching decisions (performance evaluated by the coach, minutes played, and selection for the National Team) are influenced by player experience and maturity (anthropometric and fitness).

## 2. Materials and Methods

### 2.1. Participants

A total of 396 Under-14 years (U14) players, who were selected by the 22 regional associations for the 2019 national inter-association tournament Lopes da Silva, participated in the study. Of these players, 36 were excluded (including one entire team), either because they did not show up (N = 18) to the assessment or because they were missing some measurement in the database (N = 18). The remaining 360 were all assessed (age: 14.11 ± 0.31 years; stature: 169.50 ± 6.99 cm; body mass: 55.69 ± 7.16 kg; skeletal age: 14.85 ± 1.18 years; maturity status: 0.74 ± 1.14 years; years of practice: 7.91 ± 2.03 years).

### 2.2. Experimental Approach

A cross-sectional study was conducted. The participants were measured during the 2019 U14 Lopes da Silva tournament. The Lopes da Silva tournament is played with teams from the different districts of Portugal; during the first phase, each team played five games, with the best two classified teams playing in the finals. In the 2019 edition, 21 teams participated in the tournament. Each team had a rooster of 18 players, with all players required to play at least 90 min, and 9 substitutions could be held during each game. The measurements took place before and during the tournament. All morphologic and fitness data were collected by researchers. The measurements were scheduled during the tournament to ensure that the evaluations were conducted at least two hours before and after the games. The test battery used in the study covered maturity, morphology, and handgrip strength. Anthropometric measures were undertaken before the functional skill test.

### 2.3. Success Measurements

Three different variables were defined to obtain performance success: performance evaluation by the coach (PEC), minutes played during the tournament, and the selection for the Under-15 years (U15) National Team in the beginning of the next season. PEC consisted of rating each player on a 1 to 5 scale according to their present performance. When it comes to minutes played, tournament rules required each player to play at least 90 min of the total minutes played (300 or 360 min) by their team. Each game had two parts consisting of 30 min, so a team that participated only in the 1st phase would necessarily complete at the end of this phase 300 min of play, the two teams that reached the finals played one more game and therefore achieved a total playtime of 360 min. The variable minutes played was transformed into a minute played ratio, obtained as (Minutes played by the player)/(Total minutes played by the team). Statistical analyses were performed using the minutes played ratio, but the results presented on Table 1 were the absolute values-minutes played. Finally, the selection of players for the U15 National Team was presented as a binary variable between select or not selected.

### 2.4. Practice Experience and Training Load

Each player was asked about the number of practices per week and duration of each one. A weekly training load (min) variable was computed, resulting from the multiplication of training frequency and training duration. Years of practice were also obtained.

### 2.5. Anthropometric Measures

Body mass (kg), stature (cm), sitting height (cm), 2nd and 4th digit length, and two skinfolds (mm) (triceps, TRI and subscapular, SBS) were measured following the International Society for the Advancement of Kinanthropometry (ISAK) protocol [21]. Leg length was calculated as the difference between stature and sitting height. Body mass was measured with a Seca Vogel & Halke (Hamburg, Germany) body scale (model 761 7019009) to the nearest 0.5 kg, and stature and sitting height were measured with a Siber–Hegner anthropometric kit (Tokyo, Japan) rounded off to the nearest 0.1 cm. Skinfolds were measured using a Slim Guide caliper (Creative Health Products, Ann Arbor, MI, USA). All measurements were made by an ISAK anthropometric technician who held a Level 2 qualification. The intra-observer technical error of measurements-%TEM (and coefficient of reliability-R) were well below the accepted maximum for stature (R ≥ 0.98), with 5% for skinfolds (0.90 ≤ R ≤ 0.98) [21]. Body mass index (BMI) was calculated using the formula BMI = Body mass/Stature^2^ (kg/m^2^).

### 2.6. Chronological Age

Chronological age (CA) was calculated (in decimals) as the difference between the evaluation date and birth date. According to the Fédération Internationale de Soccer Association (FIFA) [22], age grouping follows the calendar year, so the same rationale was applied to define birth quarters: Q1—January to March; Q2—April to June; Q3—July to September; Q4—October to December.

### 2.7. Biological Maturity

Skeletal age was measured. A left hand-wrist X-ray was performed on each participant and 13 of the total hand-wrist bones were evaluated according to the Tanner–Whitehouse III method (TW3) [23].

To determine the MS of the players, the difference between skeletal age and chronological age was calculated. The players were then divided according to their MS. If MS < −1, it was considered late maturing; if −1 ≤ MS ≤ 1, it was considered average maturing; if MS > 1, it was considered early maturing [23].

Predicted stature was also calculated through the Equation [23]:

Predicted adult stature = present stature + a × RUS score + b

where

$$a=-[0.0402-0.00632\;(age-14)-0.00155\;{\left(age-14\right)}^{2}+0.00019\;{\left(age-14\right)}^{3}]$$

$$b=37.62-5.50\;(age-14)-0.799\;{\left(age-14\right)}^{2}$$

Maturity offset (MO) was also calculated using the Mirwald method [24]:

$$\begin{array}{c} MO=-9.236+[0.0002708(Leg\;length\times Sitting\;height)]\\ \;+[-0.001663(Decimal\;age\times Leg\;length)]\\ \;+[0.007216(Decimal\;age\times Sitting\;height)]\\ \;+(0.02292\times body\;mass/height\times 100) \end{array}$$

### 2.8. Strength Measures

Handgrip strength, considered a covariate of general physical ability in adolescents [25,26], was assessed with a dynamometer (Takei Physical Fitness Test, TKK 5001, GRIP-A, Tokyo, Japan). To perform a handgrip strength test, the participant was in the standing position with the arm on the side of the body, the elbow and wrist fully extended, the shoulder slightly abducted (10°), and the forearm in neutral position [26,27]. Participants performed the test with the right and left hand. For each hand, the test was performed twice, with 1 min rest between trials. The best trial was recorded.

The intraclass correlation coefficient was calculated for the handgrip tests (Table 1).

### 2.9. Statistical Analyses

Statistical analyses were performed using the Statistical Package for the Social Sciences software (SPSS software version 29.0, 241, IBM SPSS, Chicago, IL, USA). The significance level was set at 5%.

Descriptive statistics regarding morphological, fitness, and success measurements (mean and standard deviation for all variables, except for the variable selection for the U15 National Team, for which frequency and percentage are presented) were determined according to three maturational status groups (early, on-time, late).

Pearson’s Chi-square goodness-of-fit test was conducted on the total sample to assess the distribution of birth dates across birth quartiles. Additionally, Pearson’s Chi-square tests for homogeneity were performed to compare birth quartile distributions by maturation status and birth quartiles. The effect size was determined using Cramér’s V. For 2 degrees of freedom, the effect was considered small if 0.100 ≤ V < 0.300, medium if 0.300 ≤ V < 0.500, and large if V ≥ 0.500 [28]. For 3 degrees of freedom, the effect was considered small if 0.071 ≤ V < 0.212, medium if 0.212 ≤ V < 0.354, and large if V ≥ 0.354 [28]. For 6 degrees of freedom, the effect was considered small if 0.041 ≤ V < 0.122, medium if 0.122 ≤ V < 0.204, and large if V ≥ 0.204 [28].

To compare maturation status and quartiles of birth groups, Analysis of Variance (ANOVA) and Tukey post-hoc tests were performed on maturation, morphological, fitness, and success variables that met the ANOVA assumptions. The Shapiro–Wilk test was used to assess normality, while the Leven test was conducted to evaluate the homogeneity of variances. If only the assumption of homogeneity of variances was violated, Welch’s ANOVA and Games–Howell post-hoc tests were conducted. If normality was violated, Kruskall–Wallis H test was performed instead, followed by Dunn–Bonferroni post-hoc tests. Analysis of Covariance (ANCOVA), followed by Bonferroni post-hoc tests, was conducted to assess significant differences among quartiles of birth after accounting for the effect of bone age. Bone age was controlled to understand the independent effects of birth quarters distribution. Preliminary analyses were performed to verify the assumptions of normality, homogeneity of variances, and homogeneity of regression slopes. When these assumptions were not met, Quade’s nonparametric ANCOVA was applied, followed by multiple comparisons with Bonferroni correction. The effect size was calculated using partial eta-squared ($\eta_{p}^{2}$ or $\eta_{H}^{2}$) and, according to the Cohen [28] benchmarks, 0.01 ≤ $\eta_{p}^{2}$ < 0.06 indicates a small effect, 0.06 ≤ $\eta_{p}^{2}$ < 0.14 a moderate effect, and $\eta_{p}^{2}$ ≥ 0.14 a large effect.

## 3. Results

**Figure 1:**
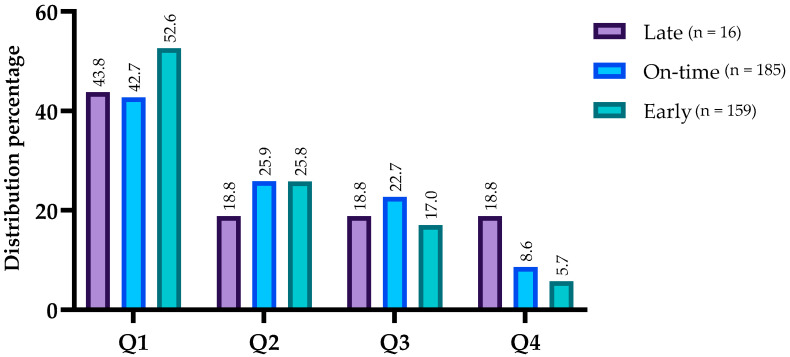
Distribution of players according to maturity status and birth quarters. Note. Q1: 1st birth quartile; Q2: 2nd birth quartile; Q3: 3rd birth quartile; Q4: 4th birth quartile.

The 360 players measured were organized according to maturation status and birth quarter (Figure 1). Considering the total sample there was a significant difference in the distribution by birth quarters (χ^2^ (3) = 113.956, *p* < 0.001, V = 0.325). Most of the sample was born on Q1 (45.8%), followed by Q2 (24.9%), Q3 (19.5%), and Q4 (7.6%). The distribution of each MS across birth quarters is similar to that described for the total sample. No differences were found when comparing the distribution of each birth quartile between the maturity status groups (χ^2^ (6) = 6.763, *p* = 0.341, V = 0.097), as shown in Figure 1.

**Figure 2:**
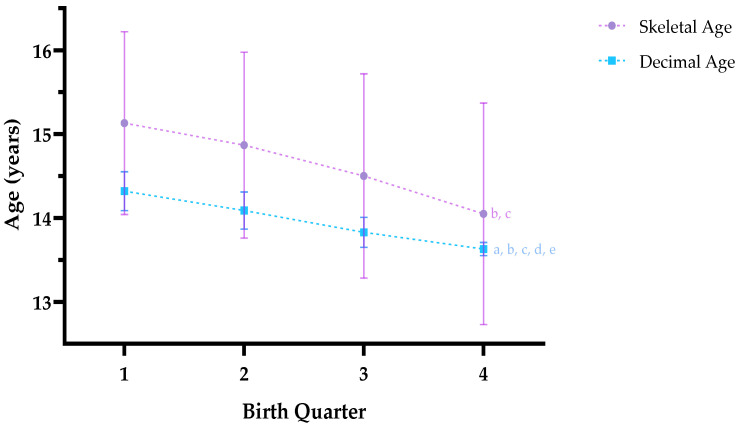
Skeletal age (mean ± SD) and Decimal age (mean ± SD) according to birth quartiles. Note. Q1: 1st birth quartile; Q2: 2nd birth quartile; Q3: 3rd birth quartile; Q4:4th birth quartile. *p* < 0.05: a, Q1 vs. Q2; b, Q1 vs. Q3; c, Q1 vs. Q4; d, Q2 vs. Q3; e, Q2 vs. Q4.

When comparing skeletal and decimal ages by birth quartiles, significant differences were found (decimal age, H (3) = 264.452, *p* < 0.001; skeletal age, H (3) = 23.560, *p* < 0.001). These differences are shown in Figure 2.

Table 2 presents the differences for each variable according to the maturation status. After controlling for years of practice and training load (ANCOVA), differences across maturation status were significant for decimal age (*p* < 0.001, $\eta_{H}^{2}$ = 0.017), the difference between bone age and decimal age (*p* < 0.001, $\eta_{H}^{2}$ = 0.782), bone age (*p* < 0.001, $\eta_{H}^{2}$ = 0.763), MO (*p* < 0.001, $\eta_{H}^{2}$ = 0.263), predicted stature (*p* < 0.001, $\eta_{H}^{2}$ = 0.203), stature (*p* < 0.001, $\eta_{H}^{2}$ = 0.152), body mass (*p* < 0.001, $\eta_{H}^{2}$ = 0.214), BMI (*p* < 0.001, $\eta_{H}^{2}$ = 0.108), sitting height (*p* < 0.001, $\eta_{H}^{2}$ = 0.276), 2nd digit length (*p* = 0.300, $\eta_{H}^{2}$ = 0.034), 4th digit length (*p* = 0.023, $\eta_{H}^{2}$ = 0.035), subscapular skinfold (*p* < 0.001, $\eta_{H}^{2}$ = 0.068), right handgrip strength (*p* < 0.001, $\eta_{H}^{2}$ = 0.194), and left handgrip strength (*p* < 0.001, $\eta_{H}^{2}$ = 0.184).

**Figure 3:**
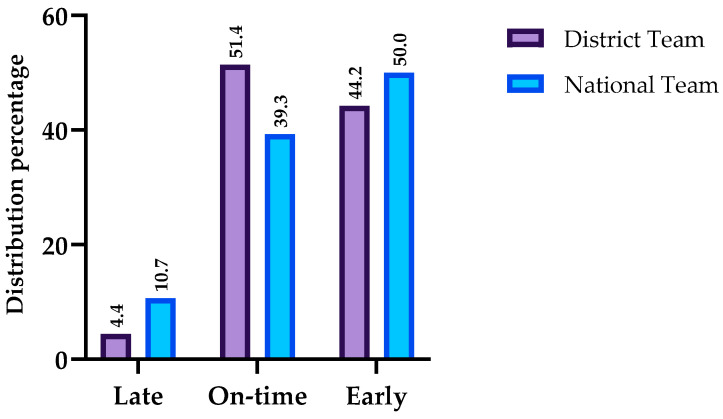
Maturity status distribution of players selected from the District and National Teams.

The players maturation status distribution for the District and National Teams are presented in Figure 3. The selected players for the National Team did not differ according to maturation status (χ^2^ (2) = 3.785, *p* = 0.140, V = 0.103).

Table 3 presents the differences for each variable according to birth quarters and in the selection for the National team (χ^2^ (3) = 10.047, *p* = 0.018, V = 0.167). After controlling for bone age (ANCOVA), differences between birth quarters were significant for decimal age (*p* < 0.001, $\eta_{H}^{2}$ = 0.564), MO (*p* < 0.001, $\eta_{p}^{2}$ = 0.090), and years of practice (*p* < 0.001, $\eta_{H}^{2}$ = 0.008). Decimal age is different between all the birth quarters. MO is different between the 1st quarter and the 3rd and 4th quarters as well as between the 2nd and 4th quarters. Years of practice are different between the 2nd and 3rd quarters.

The players selection for the National Team differed according to birth quartiles (χ^2^ (3) = 10.047, *p* = 0.018). From the 167 players born in Q1, 7.7% have been selected; from the 92 born in Q2, 14.1% have been selected; from the 72 born in Q3, 2.8% have been selected; from the 28 born in Q4, none have been selected for the National Team.

## 4. Discussion

This study evaluated the best U14 soccer players from across Portugal during the Lopes da Silva tournament. The aim was to investigate the impact of relative age and maturation status on anthropometric characteristics, handgrip strength, training load, years of practice, and coaches’ decisions regarding playing time and selection for the U14 Regional Teams and the U15 Portugal National Team. Additionally, the study sought to confirm whether the interrelationship between relative age and maturation status observed in Portuguese elite teams remains consistent with findings from 2015 [16].

In this study, most players were born in Q1 (46.4%) and Q2 (25.6%), regardless of maturational status, while only 27.8% were born in Q3 and Q4. These findings aligned with previous research [16,29,30].

The 14–15 age range is widely recognized as a critical period for talent identification in soccer, although the RAE in soccer is evident as early as U9 [13] and has been observed in both elite and non-elite settings [5,8,11,30,31,32,33,34,35,36].

Differences between chronologically older and younger players can be either mitigated or amplified by individual maturity variations [34,37]. Around the age of 14, morphological and functional disparities between early and late maturers become more pronounced [15,38], significantly influencing coaches’ selection decisions. Our results support this pattern, as only 16 players (4.44%) out of 360 selected for District Teams were classified as late-maturing.

Although RAE and maturation should be accounted separately [15], they yielded similar differences when comparing quartile groups. Predicted height, BMI, second digit length, and subscapular skinfold varied across maturation groups, but showed no significant differences between quartiles. Consistent with Eskandarifard et al. [39] and Goto et al. [40], early maturing players had greater trunk length and body mass. Similarly, Toselli, Mauro, Grigoletto, Cataldi, Benedetti, Nanni, Di Miceli, Aiello, Gallamini, Fischetti, and Greco [32] found that early-maturing Italian players were taller, heavier, and had better body composition than their peers. Late-maturing players were shorter, lighter, had smaller trunk length, lower left handgrip strength, and played fewer minutes than their peers. They also had lower BMI, digit lengths, subscapular skinfold, and right handgrip strength compared to early maturers. These findings contrast with Massa, Moreira, Costa, Lima, Thiengo, Marquez, Coutts, and Aoki [8] who found no maturation-based differences among Brazilian soccer players.

Bone age varied significantly by birth quartile, with Q1 players having a greater mean bone age difference (0.83 years) than Q4 players (0.42 years). Despite a six-month chronological age gap between Q1 and Q4 players, their skeletal age difference averaged 12 months, suggesting maturity-driven morphological and fitness differences. When bone age was considered, these differences became non-significant. This supports findings that Q3 and Q4 players can catch up in body dimensions and performance, potentially reversing RAE at senior levels, where later-quartile births are more common among professionals [2,5,31,41].

When relating bone age and birth quartiles or MS and birth quartiles, we found different results from Fragoso, Massuça, and Ferreira [16]. This study showed that in all the birth quartiles, there was a preference for more mature players, while Fragoso, Massuça, and Ferreira [16] found that this effect was especially reduced in Q3 and Q4. This means that coaches selected for the District Teams more mature players independently of their birth quarter. This result may also show a certain conceptual evolution. The general awareness of the increased number of senior and professional players born in Q3 and Q4 may have boosted the number of registered and not excluded players born later in the year as well as late maturers in the development teams, allowing coaches to choose the most mature ones as indicated in the other quartiles.

Experience, often measured as years of practice, has been considered a main and distinctive factor by Deprez, et al. [42]. However, years of practice and training load results were not significantly different between maturation status. The studied players reported a mean value of 7.9 years of soccer practice independently of their maturation status. Players averaged 7.9 years of practice, irrespective of maturity. Players born in Q1 and Q2 had slightly more practice time than those in Q3 and Q4, with a significant difference between Q2 (8.01 years) and Q3 (7.34 years). Training loads were consistent across groups at approximately 285 min per week.

Coaches’ decision making was analyzed through the minutes played and U15 National Team selection. A clear maturational bias emerged, as players with greater physical and functional abilities—typically early maturers—presented more playing time [2,16]. Despite lacking formal maturity status data, it can be assumed that coaches favored on-time and early maturers, likely due to their physical, functional, and cognitive advantages. Late maturers averaged at 80 min, falling short of the tournament’s minimum 90 min rule. This discrepancy may also result from rules violations or higher injury risks for late-maturing players competing against more mature peers [30]. Additionally, birth quarter had little impact on playing time, though Q1 players played 30 min more on average.

The coaches’ selection for the National Team showed a maturational bias. This result is clear since only three late-maturing subjects (10.7%) were chosen while the majority of players chosen for the National Team were either on-time (39.3%) or early (50%). Considering the small number of late-maturing players, just sixteen, we hypothesized that the preliminary selection for the District Teams had already left behind potentially good players, but they were not chosen because of their maturation [43]. As pointed out through our results, late maturing players are shorter, thinner, and have fewer opportunities to play, meaning that they were probably chosen because they excel in other areas, such as technical ability or tactical knowledge. Another aspect not to be forgotten during the selection process, according to Konarski, Krzykała, Skrzypczak, Nowakowska, Coelho, Cumming, and Malina [4], is the predicted adult height, which, in this study, was greater in late-maturing players, while more mature players were closer to their potential height [40]. Massa, Moreira, Costa, Lima, Thiengo, Marquez, Coutts, and Aoki [8] asserted that the prevalence of RAE in the National Teams depends on the presence of RAE in the semi-elite teams from which the selection takes place. The players selected for the National Team were all born in the first and second quartile except for two; in other words, 26 of the 28 selected players belong to Q1 and Q2.

We are, undoubtedly, facing two decisive conditions that influence coaches in the long, medium, and short term. The consequences of RAE and maturation biases are significant. Late-maturing athletes face limited playing opportunities in tournaments like Lopes da Silva and beyond. Without alternative training or competition strategies, these players are at risk of dropping out. Conversely, players from Q1 and Q2 were selected for the U15 National Team, likely due to their size and maturity. This suggests that their success may be largely dependent on their physical attributes, despite coaches emphasizing a holistic evaluation that includes technical and tactical skills. Efforts to mitigate RAE and maturation disparities have included grouping players by stature, weight, biological age, or MS (bio-banding) [44,45,46,47] using talent identification strategies [48] like dressing the athlete in a colored vest depending on their state of maturity, changing the start dates of the sport season [49,50,51], rotating the cutoff dates [49,52], and constructing a narrower time sports categories or others [53]. Recently, Helsen, Thomis, Starkes, Vrijens, Ooms, MacMaster, and Towlson [30] proposed reallocating youth players based on Estimated Developmental (ED) age, calculated from mean stature growth curves. This method effectively reduces RAE and maturation biases [13].

This study has limitations, as it only analyzed tournament data within a narrow age range, making the findings inapplicable to other age groups. Players underwent prior club selection, which likely influenced District Team choices. The practical experience variable only accounted for soccer-specific time and load, failing to capture other sports or daily activities. Injuries were not assessed, which could have clarified the reduced playtime of late maturers. Given the sample’s bias toward early and on-time maturers, further research is needed to explore maturation, RAE, and the point at which late maturers are overlooked in selection processes.

## 5. Conclusions

Undoubtedly, maturation status and RAE are two major causes of a Portuguese coach’s decision-making, but as the morphologic and functional differences between players born in different quartiles were no longer significant after removing bone age effect, it can be assumed that coach decision about the selection to the National Team is influenced by the maturational status. This situation has remained almost unchanged since 2015, although currently athletes born in the third and fourth quartile and chosen to play on elite or semi-elite teams are equally more mature. Finally, the squad of the District Teams presented a greater maturational bias (4%) than the group of players chosen to join the National Team (10%).

Therefore, to ensure fair talent selection, soccer clubs, associations, and federations should implement bio-banding, track maturation bias, and promote long-term development programs for young players. Coaches and scouts and managers should adopt holistic assessment criteria, provide appropriate training for players of different maturity status, and support flexible competition structures. These strategies will create a more inclusive and development-focused talent identification system in soccer.

## Figures and Tables

**Table 1:** Intraclass correlation statistics for intra-rater reliability for the physical tests.

	ICC ^b^	95% CI		F Test with True Value 0			
		Lower Bound	Upper Bound	Value	df1	df2	*p*
HG right hand	0.867	0.840	0.891	14.092	359	359	<0.001
HG left hand	0.886	0.862	0.906	26.567	359	359	<0.001

Note: ^b^ ICC: Intraclass correlation based on a consistency, two-way mixed model; CI: Confidence interval; ICC: Intraclass correlation coefficient; HG: Handgrip strength.

**Table 2:** Comparison of maturational, morphological, fitness, and success variables of U14 soccer players according to maturation status.

		ANOVA				
		Maturation Status			*p*	Effect Size
Variables	Total	Late (*n* = 16)	On-Time (*n* = 185)	Early (*n* = 159)		
Decimal age (years) ^b^	14.11 ± 0.31	14.13 ± 0.32	14.01 ± 0.31	14.15 ± 0.29	0.065	0.017
Maturity						
BA and DA difference (years) ^b^	0.74 ± 1.14	−1.82 ± 1.15 ^†^	0.06 ± 0.53 ^†^	1.79 ± 0.44 ^†^	<0.001	0.778
Bone age (years) ^b^	14.85 ± 1.18	12.27 ± 1.35 ^†^	14.14 ± 0.55 ^†^	15.94 ± 0.48 ^†^	<0.001	0.749
Maturity offset (years) ^a^	0.55 ± 0.57 ^†^	−0.31 ± 0.56 ^†^	0.38 ± 0.52 ^†^	0.84 ± 0.45 ^†^	<0.001	0.263
Predicted stature (cm) ^b^	178.09 ± 7.19	178.08 ± 7.66	181.09 ± 6.27 ^‡^	174.58 ± 6.60	<0.001	0.218
Morphological						
Stature (cm) ^b^	169.50 ± 6.99	159.07 ± 9.29 ^†^	168.43 ± 6.75 ^†^	171.75 ± 5.80 ^†^	<0.001	0.115
Body mass (kg) ^c^	55.69 ± 7.17	46.57 ± 8.83 ^†^	53.51 ± 5.96 ^†^	59.13 ± 6.56 ^†^	<0.001	0.217
BMI (kg/m^2^) ^b^	19.34 ± 1.85	18.28 ± 2.17 ^‡^	18.85 ± 1.72 ^‡^	20.02 ± 1.76	<0.001	0.146
Sitting height (cm) ^a^	88.39 ± 3.82	81.78 ± 3.45 ^†^	87.28 ± 3.49 ^†^	90.23 ± 2.95 ^†^	<0.001	0.275
Leg length (cm) ^b^	81.16 ± 4.55	77.29 ± 6.32	81.15 ± 4.79	81.52 ± 3.92	0.066	0.015
2nd Digit length (cm) ^b^	7.16 ± 0.44	6.80 ± 0.72 ^‡^	7.14 ± 0.40	7.21 ± 0.43	0.035	0.019
4th Digit length (cm) ^b^	7.30 ± 0.43	6.94 ± 0.63	7.29 ± 0.40 ^§^	7.34 ± 0.43 ^§^	0.039	0.018
2D/4D ratio ^b^	0.98 ± 0.03	0.98 ± 0.03	0.98 ± 0.03	0.98 ± 0.04	0.647	0.002
Tricipital skinfold (mm) ^b^	7.88 ± 2.71	9.7 ± 5.66	7.65 ± 2.51	7.97 ± 2.44	0.201	0.009
Subscapular skinfold (mm) ^b^	6.29 ± 0.43	6.5 ± 4.79 ^‡^	5.81 ± 1.51 ^‡^	6.85 ± 1.93	<0.001	0.131
Father stature (cm) ^c^	175.62 ± 5.81	175.33 ± 8.30	175.96 ± 5.56	175.27 ± 5.84	0.565	0.003
Mother stature (cm) ^a^	163.73 ± 5.78	165.80 ± 6.27	163.98 ± 5.95	163.31 ± 5.52	0.283	0.007
Fitness						
Right handgrip strength (N) ^b^	32.66 ± 6.10	25.30 ± 5.42 ^‡^	30.80 ± 5.10 ^‡^	35.50 ± 5.85	<0.001	0.187
Left handgrip strength (N) ^b^	30.66 ± 5.63	24.02 ± 4.98 ^†^	28.99 ± 4.50 ^†^	33.23 ± 5.66 ^†^	<0.001	0.167
Experience						
Years of practice (#) ^b^	7.91 ± 2.03	7.40 ± 2.17	8.16 ± 1.81	7.65 ± 2.22	0.111	0.014
Training load (min) ^b^	284.26 ± 75.28	300.00 ± 91.94	281.25 ± 67.69	285.02 ± 80.88	0.783	0.048
Success						
PEC ^b^	3.25 ± 0.78	2.93 ± 0.88	3.23 ± 0.76	3.29 ± 0.79	0.524	0.004
Minutes played (min) ^b^	152.77 ± 69.88	80.20 ± 60.36	151.11 ± 66.62 ^§^	161.31 ± 70.51 ^§^	<0001	0.048
Selection for the National Team (frequency) ^d^	28 (7.8%)	3 (18.8%)	11 (5.9%)	14 (8.8%)	0.140	0.103

Note. BA: bone age; DA: decimal age; PEC: performance evaluation by the coach; ^a^ One-way ANOVA; ^b^ Kruskall–Wallis ^c^ One-way ANOVA with Welch correction; ^d^ Pearson’s Chi-squared test for homogeneity; ^†^ significant difference between all groups; ^‡^ significant difference with early maturation status; ^§^ significant difference with late maturation status.

**Table 3:** Comparison of maturational, morphological, fitness, and success variables of U14 soccer players according to birth quartile.

	ANOVA						ANCOVA (Controlling Bone Age)					
	Quartile of Birth				*p*	Effect Size	Quartile of Birth				*p*	Effect Size
Variables	1st (*n* = 167; 46.4%)	2nd (*n* = 92; 25.6%)	3rd (*n* = 72; 20%)	4th (*n* = 28; 7.8%)			1st (*n* = 167)	2nd (*n* = 92)	3rd (*n* =72)	4th (*n* = 28)		
Decimal age (years) ^b^	14.32 ± 0.20	14.09 ± 0.22	13.83 ± 0.18	13.63 ± 0.08	<0.001	0.737	14.32 ± 0.19 ^†^	14.09 ± 0.19 ^†^	13.84 ± 0.20 ^†^	13.64 ± 0.20 ^†^	<0.001	0.564
Maturity												
BA and DA difference (years) ^b^	0.80 ± 1.08	0.79 ± 1.13	0.67 ± 1.22	0.43 ± 1.31	0.663	0.004	-	-	-	-	-	-
Bone age (years) ^b^	15.15 ± 1.08 ^£¥^	14.87 ± 1.11 ^£^	14.50 ± 1.22 ^‡^	14.05 ± 1.31 ^‡§^	<0.001	0.066	-	-	-	-	-	-
Maturity offset (years) ^a^	0.72 ± 0.53 ^¥£^	0.58 ± 0.52 ^¥£^	0.31 ± 0.51 ^‡§£^	−0.03 ± 0.55 ^‡§¥^	<0.001	0.161	0.65 ± 0.43 ^¥£^	0.58 ± 0.41 ^£^	0.41 ± 0.42 ^‡^	0.20 ± 0.42 ^‡§^	<0.001	0.090
Predicted stature (cm) ^b^	177.01 ± 6.83	178.71 ± 7.69	179.20 ± 6.91	179.47 ± 7.77	0.093	0.018	177.67 ± 6.78	178.76 ± 6.69	178.39 ± 6.75	177.67 ± 6.81	0.400	0.005
Morphological												
Stature (cm) ^b^	170.15 ± 6.98 ^£^	170.43 ± 6.38 ^£^	168.42 ± 6.99	165.41 ± 7.67 ^‡§^	0.004	0.037	169.40 ± 6.27	170.38 ± 6.19	169.35 ± 6.25	167.55 ± 6.30	0.186	0.013
Body mass (kg) ^a^	56.58 ± 7.26 ^£^	56.14 ± 6.54 ^£^	54.99 ± 7.09 ^£^	50.79 ± 7.15 ^‡§¥^	<0.001	0.046	55.65 ± 6.03	56.07 ± 5.94	56.16 ± 6.00	54.44 ± 6.05	0.721	0.013
BMI (kg/m^2^) ^b^	19.51 ± 2.01	19.30 ± 1.75	19.33 ± 1.67	18.47 ± 1.47	0.071	0.020	19.36 ± 1.75	19.29 ± 1.74	19.53 ± 1.75	18.92 ± 1.77	0.423	0.007
Sitting height (cm) ^a^	88.88 ± 3.73 ^£^	88.70 ± 3.71 ^£^	87.59 ± 3.69	85.99 ± 4.04 ^‡§^	<0.001	0.048	88.32 ± 3.07	88.66 ± 3.03	88.27 ± 3.05	87.54 ± 3.09	0.713	0.008
Leg length (cm) ^b^	81.27 ± 4.99 ^£^	81.73 ± 3.94 ^£^	80.82 ± 4.14	79.42 ± 4.40 ^‡§^	0.028	0.025	81.08 ± 4.52	81.71 ± 4.46	81.08 ± 4.50	80.00 ± 4.55	0.098	0.009
2nd Digit length (cm) ^b^	7.22 ± 0.44	7.12 ± 0.38	7.14 ± 0.48	6.97 ± 0.46	0.058	0.021	7.35 ± 0.43	7.26 ± 0.42	7.29 ± 0.42	7.08 ± 0.43	0.296	0.012
4th Digit length (cm) ^b^	7.35 ± 0.44 ^£^	7.34 ± 0.36	7.29 ± 0.47	7.08 ± 0.42 ^‡^	0.039	0.023	7.33 ± 0.43	7.26 ± 0.41	7.32 ± 0.42	7.14 ± 0.42	0.171	0.016
2D/4D ratio ^b^	0.98 ± 0.04	0.98 ± 0.03	0.98 ± 0.03	0.99 ± 0.03	0.871	0.002	0.98 ± 0.04	0.98 ± 0.04	0.98 ± 0.03	0.99 ± 0.04	0.860	0.003
Tricipital skinfold (mm) ^b^	7.88 ± 2.58	7.78 ± 3.19	8.08 ± 2.66	7.74 ± 1.67	0.463	0.007	7.90 ± 2.75	7.79 ± 2.71	8.03 ± 2.74	7.64 ± 2.77	0.419	0.002
Subscapular skinfold (mm) ^b^	6.72 ± 1.99	6.43 ± 2.48	6.14 ± 1.45	5.59 ± 1.17	0.200	0.013	6.30 ± 1.97	6.42 ± 1.95	6.26 ± 1.96	55.88 ± 1.98	0.680	0.005
Father stature (cm) ^b^	175.34 ± 5.45	176.01 ± 6.11	175.42 ± 6.16	176.50 ± 6.10	0.835	0.002	175.37 ± 5.92	176.01 ± 5.82	175.37 ± 5.88	176.39 ± 5.94	0.906	0.004
Mother stature (cm) ^a^	164.05 ± 5.91	163.70 ± 6.08	162.82 ± 5.46	164.43 ± 4.73	0.450	0.007	164.14 ± 5.85	163.70 ± 5.77	162.68 ± 5.82	164.10 ± 5.88	0.238	0.009
Fitness												
Right handgrip strength (N) ^a^	34.03 ± 6.38 ^§¥£^	31.81 ± 5.31 ^‡^	31.70 ± 5.74 ^‡^	29.56 ± 5.92 ^‡^	<0.001	0.054	33.29 ± 5.12	31.76 ± 5.05	32.68 ± 5.09	31.79 ± 5.14	0.099	0.017
Left handgrip strength (N) ^a^	32.07 ± 5.82 ^§¥£^	29.84 ± 5.00 ^‡^	29.54 ± 5.25 ^‡^	27.65 ± 5.42 ^‡^	<0.001	0.065	31.41 ± 4.76	29.80 ± 4.70	30.41 ± 4.74	29.63 ± 4.79	0.454	0.024
Experience												
Years of practice (years) ^b^	8.06 ± 2.11	8.01 ± 2.00	7.47 ± 1.96	7.79 ± 1.73	0.083	0.019	8.12 ± 2.03	8.01 ± 2.00 ^¥^	7.39 ± 2.03 ^§^	7.60 ± 2.05	0.032	0.020
Training load (min) ^b^	280.03 ± 71.21	287.85 ± 79.79	286.67 ± 80.50	283.93 ± 63.89	0.736	0.004	281.41 ± 76.67	287.87 ± 75.64	286.42 ± 76.37	283.37 ± 77.09	0.785	0.001
Success												
PEC ^b^	3.74 ± 0.78	3.73 ± 0.80	3.68 ± 0.71	3.50 ± 0.75	0.475	0.007	3.74 ± 0.78	3.73 ± 7.67	3.68 ± 0.77	3.50 ± 0.78	0.369	0.008
Minutes played (min) ^b^	162.05 ± 69.48	144.98 ± 68.19	148.29 ± 73.70	136.71 ± 63.93	0.116	0.016	158.13 ± 69.10	144.73 ± 68.19	152.80 ± 68.84	146.94 ± 69.49	0.386	0.007
Selection for the National Team (frequency) ^d^	13 (46.4%)	13 (46.4%)	2 (7.1%)	0 (0%)	0.018	0.167	-	-	-	-	-	-

Note. BA: bone age; DA: decimal age; PEC: performance evaluation by the coach; ^a^ One-way ANOVA; ^b^ Kruskall–Wallis; ^d^ Pearson’s Chi-squared test for homogeneity; ^†^ significant difference between all groups; ^‡^ significant difference with the 1st quartile; ^§^ significant difference with the 2nd quartile; ^¥^ significant difference with the 3rd quartile; ^£^ significant difference with the 4th quartile.

## Data Availability

The datasets generated and/or analyzed during the current study are not publicly available due to privacy/ethical restrictions and are only available from the corresponding author upon reasonable request.

## Abbreviations

The following abbreviations are used in this manuscript:

CA	Chronological age
MO	Maturity offset
MS	Maturation Status
PEC	Performance Evaluation by the Coach
Q1	1st birth quarter
Q2	2nd birth quarter
Q3	3rd birth quarter
Q4	4th birth quarter
RAE	Relative Age Effect
TW3	Tanner–Whitehouse III method
U14	Under 14
U15	Under 15
